# The effectiveness of Afrislum's expert client-delivered group brief cognitive behavioural therapy model in reducing HIV self-stigma among pregnant adolescent girls and young women living in urban informal settlements of Kampala, Uganda: a quasi-experimental difference-in-differences analysis

**DOI:** 10.11604/pamj.2026.53.148.43474

**Published:** 2026-04-01

**Authors:** Susan Babirye, Shafiq Kawooya, Jennipher Florence Namuli, Christopher Oundo, Cecilia Kihara

**Affiliations:** 1 Department of Government Studies, Uganda Management Institute, Kampala, Uganda,; 2 Department of Research and Evaluation, Afrislum Uganda, Kampala, Uganda,; 3 Department of Health Policy and Planning, Makerere University School of Public Health, Kampala, Uganda,; 4 Department of Programs, Afrislum Uganda, Kampala, Uganda,; 5 Directorate of Public Health and Environment, Kampala City Council Authority, Kampala, Uganda,; 6 ViiV Healthcare, London, United Kingdom

**Keywords:** HIV self-stigma, expert client, cognitive behavioral therapy, difference-in-differences, people living with HIV, slums, adolescent girls, Uganda

## Abstract

**Introduction:**

cognitive behavioural therapy (CBT), a psychotherapeutic treatment, has been reported to effectively reduce self-stigma among people living with HIV; however, to date, there are barely any studies that have tested its effectiveness when delivered by lay counsellors. This study assessed the effectiveness of the expert client-delivered group CBT in reducing self-stigma among HIV positive pregnant adolescent girls and young mothers (AGYM) living in urban informal settlements of Kampala, Uganda.

**Methods:**

this was a quasi-experimental pre-post-test study of 253 pregnant AGYM attending Elimination of Mother-to-Child Transmission of HIV (EMTCT) and Early Infant Diagnosis (EID) clinics at four public health facilities serving the urban poor in Kampala, Uganda. In total, 142 participants in the treatment group were assigned to a 10-session CBT group, while 111 participants in the control group continued receiving treatment as usual. A difference-in-differences (DiD) analysis was conducted.

**Results:**

the DiD analysis demonstrated that the expert client delivered group CBT intervention showed a significant change/reduction of -1.121 percentage points (pp) in HIV self-stigma between baseline and endline, with P<0.001, after controlling for differences in various demographic variables and confounders between the intervention and control groups.

**Conclusion:**

this study provides evidence for the effectiveness of the expert-client-delivered group CBT HIV self-stigma reduction model among AGYM in Uganda. The use of peer-to-peer group CBT may be a promising approach for increasing access to psychosocial services in resource-limited settings like Uganda, where trained psychologists and mental health counsellors are few and psychosocial services are expensive.

## Introduction

HIV self-stigma, also known as internalized stigma, remains a major public health concern in Uganda, where 1.4 million people are living with HIV [1]. Stigma and discrimination against people with HIV/AIDs is high (54%) according to a survey by NAFOPHANU, 2015. HIV self-stigma has been documented to have substantial negative impacts across the HIV care continuum, from HIV diagnosis to linkage and retention into care, and viral load suppression. Self-stigma not only affects mental health [2] but also hampers uptake of HIV testing, prevention, and treatment services, including life-saving antiretroviral therapy (ART), thus resulting in serious epidemiological consequences. It has also been linked with HIV medication adherence [3].

In Uganda, one in every four new infections among women 15-49 years occurs in young women aged 15-24 years [4]. The prevalence of HIV among sexually active youths living in the slums of Kampala, Uganda, was at 13.9 percent [5] compared to 6.4 percent in the general adult population of Uganda. Pregnant Adolescent Girls and Young mothers (AGYM) living with HIV in slums are particularly susceptible to self-stigma because they are poorly served by the health system, and face the dual burden of living with HIV, adolescence, and early motherhood challenges. Evidence shows that young people living with HIV/AIDS are more vulnerable to HIV related stigma [6]. The vulnerability to self-stigma among pregnant AGYM living with HIV in slums is worsened by social and economic marginalization [7], as well as the rapid physical and psychosocial transitions. Adolescent girls and young mothers’ situation is even more pronounced by the structural vulnerabilities that characterize slums. Adolescent girls and young mothers also suffer unjustified social blame for early pregnancy [8]. Their situation is further intensified by the general lack of youth-friendly programmes to enable AGYM to navigate through HIV-related challenges [9].

Available evidence indicates that psychosocial interventions such as cognitive-behavioral therapy (CBT) are effective in reducing self-stigma among people living with HIV [10]. Cognitive-behavioral therapy is a talk therapy that works by helping a client manage their current problem by changing the way they think and behave, hence improving their well-being. Cognitive-behavioral therapy (CBT) uses cognitive and behavioral methods to challenge dysfunctional beliefs. It also promotes more realistic and adaptive ways of thinking to bring about emotional and behavioral change [11]. Cognitive-behavioral therapy has been reported as an effective depression treatment [12-14]. However, the therapy has been mainly delivered by expert psychologists or mental health counselors, and these are inaccessible, particularly in resource-limited settings [15]. A systematic review of psychosocial interventions delivered by non-specialists found them to be beneficial to recipients [16]. Literature shows that lay counselor-delivered therapies should, however, be provided within parameters of adequate training and supervision from a specialist [15,17].

Despite the promising evidence, there is barely any studies or projects that have implemented CBT using lay counselors yet in resource-limited settings like Uganda, where psychologists, mental health counselors or psychotherapists, and psychological support services are scarce and costly. The present study aimed to pilot and evaluate the effectiveness of a 10-session group CBT intervention, delivered by experts and clients, at selected public health facilities serving the urban poor in Kampala. The goal was to determine if this approach reduces HIV self-stigma among among HIV positive pregnant AGYM living in urban informal settlements of Kampala, Uganda.

## Methods

**Study design:** this study employed a quasi-experimental pre-post-test research design with a control arm. A quasi-experimental design is frequently used for the estimation of causal relationships in settings where randomized controlled trials are infeasible or unethical [18]. A difference-in-differences (DiD) analysis was used to compare outcome changes over time between the intervention and control groups to measure the impact of the intervention [19]. The primary aim of this study was to evaluate the effectiveness of a 10-session group CBT intervention, delivered by experts and clients, at selected public health facilities serving the urban poor in Kampala. The goal was to determine if this approach reduces HIV self-stigma among among HIV positive pregnant AGYM living in urban informal settlements of Kampala, Uganda. The study involved pregnant AGYM (15-24 years) living with HIV and attending Prevention of Mother-to-Child Transmission and Early Infant Diagnosis (PMTCT/EID) services at the four public health facilities (Kitebi H/C III, Kawaala H/C III, Kiswa H/C III, and Kisenyi H/C IV) serving the urban poor in Kampala, Uganda. Kitebi, Kawaala, and Kiswa H/C IIIs were the intervention facilities, while Kisenyi H/C IV was the nonintervention/control facility. The primary outcome of this study is the change in the overall HIV self-stigma score from baseline to endline, measured using the adapted HIV Stigma Scale and estimated using a difference-in-differences (DiD) model comparing the intervention and control groups. The secondary outcomes include changes in the four sub-domains of HIV self-stigma derived from the scale: personalized stigma, disclosure concerns, negative self-image, and public attitudes toward people living with HIV.

**Description of Afrislum's expert client delivered group cognitive-behavioral therapy for HIV self-stigma reduction model:** Afrislum Uganda is an indigenous, urban-poor-focused, non-profit organization committed to implementing high-quality evidence-based, holistic approaches that address both human, social, and economic assets of an individual living in informal settlements, the slums. The CBT intervention evaluated in this paper was implemented within Afrislum-supported public health facilities serving the urban poor populations in Kampala, Uganda. Within the supported facilities, study participants were recruited from the PMTCT and EID clinics. All study participants continued receiving the usual facility-based PMTCT/EID care, including disease management provided to all HIV patients in Uganda. In the intervention group, the usual care was supplemented with Afrislum´s expert client-delivered group brief CBT for HIV self-stigma reduction.

This psychosocial model consisted of 10 different but interrelated sessions developed to address the underlying negative experiences of HIV positive pregnant adolescent girls and young mothers. The sessions included rapport building and introduction to CBT for HIV self-stigma, working with negative thoughts, self-defeating beliefs, destructive behaviors, stress and stress management, health promotion, goal setting, life skills training, building and maintaining social support networks, wrap up session. At each health facility, four expert clients were selected in consultation with the EMTCT/EID clinic staff. Expert clients were purposively selected from stable ART patients already engaged in peer-support roles within PMTCT clinics. Selection emphasized adherence history, communication skills, and willingness to support peers. Expert clients were people living with HIV (PLWH) who were stable on ART and using their experience and skills to help other PLWH achieve better treatment outcomes [20,21]. All selected expert clients received a 7-day structured training on CBT principles, stigma reduction, and group facilitation, followed by refresher training during implementation. The trained expert clients offered monthly group CBT sessions to HIV positive AGYM living in the slums of Kampala to facilitate change in their conception of HIV, sense of self-worth, and to empower them with more adaptive ways of thinking and dealing with their experience of self-stigma. Each expert was assigned a cohort of 10-12 participants to engage 10 times throughout their PMTCT journey. The beneficiaries were identified by research assistants from PMTCT clinics (at baseline) using a brief (six-item questionnaire) screening tool excerpt from the internalized stigma scale [22]. In the sessions, AGYMs were challenged to change their way of thinking and try new behaviors. After each group session, they were given homework to facilitate change from one session to the next [23].

**Study site:** this study was conducted in Kampala, Uganda. Kampala is Uganda´s capital city. It is in the Central region of Uganda. Kampala district has a population of 1,516,210 people, with a population growth rate of 2.02% per annum [24]. As the population grows and urbanization increases, there has been an increase in the number of informal settlements in the city. It is estimated that these make up at least a quarter of the city's total area, covering roughly 60% of the city's total population [25]. Kampala has five divisions, including Central, Makindye, Kawempe, Nakawa, and Rubaga divisions, with thirteen government health facilities, including four national referral hospitals (Mulago, Butabika, Kawempe, and Kiruddu); one regional referral hospital (Naguru); one health center IV (Kisenyi); and seven health center IIIs (Kisugu, Kiswa, Bukoto, Komamboga, Kitebi, Kawala, and City Hall Clinic). Specifically, this study was conducted at four public health facilities that mainly serve slum dwellers in Kampala, namely, Kitebi, Kisenyi, Kawala, and Kiswa Health centers. These facilities serve catchment populations located in informal settlements within Rubaga, Kawempe, Nakawa, and Central divisions of Kampala, where Afrislum implements community-based programming. Thus, the study facilities were purposively selected because they serve densely populated informal settlement communities and host PMTCT/EID clinics with high numbers of pregnant women living with HIV, making them suitable for piloting the expert-client delivered CBT intervention. The comparison facility (Kisenyi H/C IV), however, operates at a higher level than the intervention facilities (H/C IIIs), which may reflect differences in staffing, service capacity, and patient case-mix. Despite these differences, all facilities offered standardized PMTCT/EID services in accordance with national guidelines, thereby offering a certain degree of comparability within the core clinical pathway in which the intervention was embedded.

**Sampling design:** we used Pagano and Gauvrea´s sample size formula to determine the sample size per group (experimental and control) [26]. The study aimed to show a 10% reduction in HIV self-stigma from 24% (in an earlier study) at the end of the intervention [27]. The statistical power was 80% at a 95% level of confidence. After adjusting for a loss to follow-up of 20%, the required sample size was 141. The assumption of a 10% reduction in HIV self-stigma was used for sample size estimation and not as a predefined threshold for intervention success.

**Sampling and participant recruitment:** participants were identified and enrolled from PMTCT/EID clinics at the selected public health facilities using a facility-based screening approach. During routine clinic days, trained research assistants, in collaboration with health facility staff, screened attending clients using a brief six-item questionnaire derived from a validated internalized stigma scale to identify individuals experiencing HIV-related self-stigma. Participants who satisfied the inclusion criteria, comprising an age range of 15-24 years, confirmed HIV-positive status, and evidence of self-stigma as determined by screening, and who provided informed consent, were enrolled into the study at baseline before the initiation of the intervention. Regarding the intervention component, participants were subsequently organized into cognitive behavioural therapy (CBT) groups, while those at the comparison facility continued to receive standard care. For the end-point assessment, participants were re-invited based on their participation records, particularly attendance at CBT sessions, as delineated in the protocol.

**Data collection:** a questionnaire was designed based on the HIV self-stigma scale [22], which has a 40-item Likert-scale questionnaire covering four different aspects of HIV self-stigma. The draft questionnaire was tested at the youth corner of the non-intervention health facility, but was never used as part of the control arm, and the questionnaire was modified accordingly. The questionnaire covered participants´ characteristics, personalized stigma elements, disclosure questions, self-image questions, and public attitude information.

A facility survey was conducted from August to Sept 2021 for baseline and from August to October 2022 for endline. For the endline survey, only beneficiaries who had attended more than seven of the 10 group CBT sessions were approached to participate in the study. Trained research assistants visited the study health facilities and interviewed the eligible CBT beneficiaries using the structured questionnaire.

**Written informed consents:** written informed consents: were obtained from all participants. Married adolescents aged 15-17 years were considered emancipated and therefore self-consented. The interviews were held in a private spot at the health facility, and each interview lasted about 60 mins. Following completion of the interview, each participant received Uganda shillings 10,000 ($2.7) compensation for their time.

**Data analysis:** the basic characteristics of participants were compared between the intervention and control groups at baseline and endline. Second, the DiD analysis was conducted using the primary outcome at baseline and endline for both groups. The DiD analysis was used to evaluate the impact of the structured peer-led group CBT intervention on the individual indicators/manifestations of HIV self-stigma (personalized stigma, disclosure, negative self-image, and public attitudes). Difference-in-differences (DiD) estimates the causal effects of the treatment by assuming that in the absence of the intervention, the outcome of the intervention group would follow “parallel trends” with the outcome of the control group over time in the absence of intervention [28]. However, given that only two time points were available [baseline and endline], this assumption could not be empirically tested and is therefore treated as a maintained assumption. To enhance the analysis, a mixed-effects regression model was fitted to estimate the intervention effect, with repeated measurements nested within individuals. The model included fixed effects for time, intervention group, and their interaction, and adjusted for baseline covariates, with random effects to account for within-individual correlation. The mixed-effects framework is particularly well-suited for this study, given the longitudinal structure of the data, and provides a more robust estimation of intervention effects than a simple two-period DiD model. STATA version 15 (STATA Corporation, Texas, USA) was used for DiD analysis and the descriptive analyses.

**Ethical considerations:** we obtained approval from the Higher Degrees, Research, and Ethics Committee of the School of Public Health, Makerere University (No.942) and the Uganda National Council for Science and Technology (No. HS1488ES). The study also received administrative clearance from the Directorate of Public Health and Environment at Kampala Capital City Authority. We obtained written consent from all participants after explaining the purpose of the study. The respondents were informed about the voluntary nature of the study, and confidentiality was assured throughout the study.

## Results

**Socio-demographic characteristics of study respondents:** overall, 253 participants were enrolled at baseline, with 142 and 111 participants in the intervention and control groups, respectively. At endline, 209 participants were successfully followed up and included in the analysis, which corresponds to an overall follow-up rate of 82.6%. The endline sample comprised 103 participants in the intervention group and 106 participants in the control group. Table 1 presents the baseline socio-demographic characteristics of the participants. Overall, participants in the intervention and control groups were broadly comparable across most measured characteristics. The majority of participants in both groups were aged 20-32 years, representing 84.7% of the control group and 91.6% of the intervention group. Most participants were married or living with their partners, accounting for 83.8% of the control group and 71.1% of the intervention group.

**Table 1:** socio-demographic characteristics of participants in the expert-client delivered CBT intervention and control groups at baseline (pre-test), Kampala, Uganda, 2021-2023 (n = 253)

Characteristic	Control (n=111)	Intervention (n=142)	P-value
**Age group**			
15-19	17 (15.32%)	12 (8.45%)	0.089
20-32	94 (84.68%)	130 (91.55%)	
**Marital status**			
Divorced/separated/widowed	4 (3.60%)	6 (4.23%)	0.049
Married/living together	93 (83.78%)	101 (71.13%)	
Single/never married	14 (12.61%)	35 (24.65%)	
**Religion**			
Catholic	36 (32.43%)	46 (32.39%)	0.095
Islam	29 (26.13%)	29 (20.42%)	
Other religion	20 (18.02%)	16 (11.27%)	
Pentecostal	6 (5.41%)	20 (14.08%)	
Protestant	20 (18.02%)	31 (21.83%)	
**Education level**			
No education	0 (0.00%)	6 (4.23%)	0.007
Primary completed	21 (18.92%)	13 (9.15%)	
Secondary completed	0 (0.00%)	7 (4.93%)	
Some primary	33 (29.73%)	42 (29.58%)	
Some secondary	53 (47.75%)	72 (50.70%)	
Tertiary	4 (3.60%)	2 (1.41%)	
**Tribe**			
Baganda	48 (45.71%)	50 (46.30%)	0.375
Bakiga	6 (5.71%)	5 (4.63%)	
Banyakole	16 (15.24%)	20 (18.52%)	
Banyarwanda	5 (4.76%)	8 (7.41%)	
Banyoro	3 (2.86%)	2 (1.85%)	
Basoga	11 (10.48%)	3 (2.78%)	
Others	16 (15.24%)	20 (18.52%)	

A large proportion of participants in both groups reported some secondary education (47.8% in the control group and 50.7% in the intervention group). Catholics constituted the largest religious group among participants, followed by Muslims and Protestants. Ethnically, the Baganda formed the largest group in both arms of the study. Save for the minor differences observed in the marital status (p = 0.049) and education level (p = 0.007) between the groups, the overall distribution of baseline characteristics was broadly similar between the intervention and control groups.

**Figure 1:**
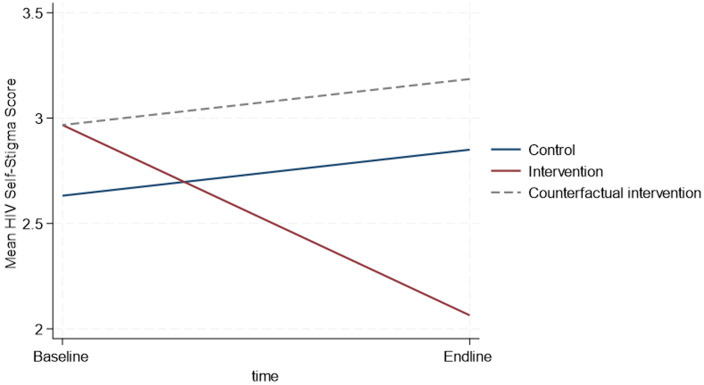
change in mean HIV self-stigma scores over time among participants in the expert-client delivered CBT intervention and control groups, Kampala, Uganda

**Effects of the intervention on the HIV self-stigma from the DiD analysis:** Table 2 presents change/effects of the intervention on the primary outcome variable (overall HIV self-stigma score) and secondary outcomes (self-stigma constructs) between baseline and endline across both the intervention and control groups. The mean HIV self-stigma score at baseline was 2.967 in the intervention group and 2.632 in the control group. At endline, mean self-stigma scores declined to 2.065 in the intervention group but increased slightly to 2.851 in the control group. This corresponds to a mean reduction of -0.902 points in the intervention group compared with an increase of 0.219 points in the control group over the study period. The resulting difference-in-differences estimate indicates that the intervention reduced overall HIV self-stigma by 1.121 points between baseline and endline (p < 0.001). Figure 1 presents a graphical representation of mean HIV self-stigma scores at baseline and endline to illustrate outcome changes over time.

**Table 2:** effect of the expert-client delivered CBT intervention on HIV self-stigma among participants (AGYWs), Kampala, Uganda, 2021-2023

Outcome	Intervention baseline (a)	Intervention endline (b)	Control baseline (c)	Control endline (d)	Difference (b-a) e	Assumption (d-c) f	DiD effect (e-f)	P-value
**Overall self-stigma**	2.967	2.065	2.632	2.851	-0.902	0.219	-1.121	<0.001
**Overall self-stigma (matched sample, n=211)**	2.994	2.065	2.639	2.851	-0.929	0.212	-1.414	<0.001
**Personalized stigma**	2.737	1.838	2.052	2.386	-0.899	0.334	-1.233	<0.001
**Disclosure**	3.292	2.332	3.228	3.334	-0.960	0.106	-1.066	<0.001
**Negative self-image**	2.893	1.945	2.665	2.851	-0.948	0.186	-1.134	<0.001
**Public attitudes**	3.019	2.105	2.699	2.909	-0.914	0.210	-1.124	<0.001

Further analysis of the individual domains (manifestations) of HIV self-stigma showed significant reductions across all four components of the stigma scale. Compared with the control group, the intervention group experienced reductions of -1.233 points for personalized stigma, -1.066 points for disclosure concerns, -1.134 points for negative self-image, and -1.124 points for perceived public attitudes, with p<0.001 for all domains. These effects remained statistically significant after accounting for differences in baseline characteristics between the intervention and control groups.

**Multivariate difference-in-differences regression analysis:** Table 3 presents the multivariate difference-in-differences regression model adjusting for select socio-demographic characteristics. After adjusting for age group, education level, marital status, religion, and number of children, the expert client-delivered group cognitive behavioral therapy (CBT) intervention remained significantly associated with a reduction in HIV self-stigma scores. The interaction term representing the DiD effect shows that the intervention reduced HIV self-stigma by -1.08 points compared with the control group (95% CI: -1.24 to -0.92, p<0.001).

**Table 3:** multivariable mixed-effects regression results for HIV self-stigma among participants (AGYWs), Kampala, Uganda, 2021-2023

Variable	β coefficient	95% CI	P-value
Intervention group (vs control)	0.33	0.22 - 0.45	<0.001
Post-intervention (vs baseline)	0.21	0.08 - 0.35	0.001
Intervention x post (DiD effect)	-1.08	-1.24 - 0.92	<0.001
**Age group (Ref: 15-19)**			
20-32	-0.08	-0.21 - 0.05	0.214
**Education level (Ref: no education)**			
Primary completed	0.01	-0.18 – 0.20	0.932
Secondary completed	-0.28	-0.48 – -0.08	0.007
Some primary	0.14	-0.04 – 0.33	0.125
Some secondary	-0.06	-0.23 – 0.12	0.528
Tertiary education	-0.33	-0.58 – -0.07	0.011
**Marital status (Ref: divorced/separated/widowed)**			
Married/living together	0.07	-0.12 – 0.25	0.480
Single/never married	0.36	0.15 – 0.57	0.001
**Religion (Ref: Catholic)**			
Islam	0.14	0.03 – 0.26	0.014
Other religion	0.11	-0.03 – 0.25	0.118
Pentecostal	0.08	-0.06 – 0.22	0.285
Protestant	-0.08	-0.19 – 0.04	0.190
**Number of children**	-0.01	-0.06 – 0.04	0.670

CI: confidence interval

The coefficient for the intervention group reflects baseline differences in stigma scores between participants in the intervention and control arms before implementation of the intervention. The intervention effect is captured by the interaction term between intervention status and the post-intervention period. Using the covariates in the model, education level was associated with stigma scores. Participants who had completed secondary education (β = -0.28, p = 0.007) and those with tertiary education (β = -0.33, p = 0.011) reported significantly lower stigma scores compared to those with no education. Marital status was also associated with stigma scores, with participants who were single or never married reporting higher stigma scores compared with those who were divorced, separated, or widowed (β = 0.36, p = 0.001). Participants identifying as Muslim reported slightly higher stigma scores compared with Catholics (β = 0.14, p = 0.014). Other socio-demographic variables, including age group, primary education, some primary or secondary education, marital status (married/living together), other religious affiliations, and number of children, were not significantly associated with HIV self-stigma scores in the adjusted model.

## Discussion

To the best of our knowledge, this is the first study to evaluate the impact of a peer-led cognitive behavioral therapy for HIV self-stigma reduction in a resource-limited setting like Uganda. The study showed that the expert client delivered group CBT reduced the overall HIV self-stigma as well as all four individual indicators of self-stigma. The results show that CBT delivered by expert clients (lay counselors) significantly reduces HIV self-stigma and may increase access to psychosocial services in resource-limited settings where psychologists and psychosocial services are barely present and very expensive if sought privately.

These results may be explained by three reasons. First, the psychotherapeutic treatment (CBT) used under the tested intervention is known for its efficacy in helping people learn how to identify and change distractive or disturbing thought patterns that are typical of a positive HIV diagnosis [29]. Although not delivered by lay counselors, a similar model using CBT for internalized stigma reduction among HIV-positive women in South Africa was found to be effective at assisting HIV-positive women in dealing with HIV and internalized stigma [10]. Secondly, the tested CBT model used expert clients to deliver group sessions. Expert clients, a cadre of lay health workers living with HIV, have been recognized in literature for improving HIV treatment uptake among people living with HIV [20,30]. Expert clients were central to the success of the group CBT sessions in the current study because they deliberately and skillfully used their own experience living with HIV to help others overcome the main challenges inherent in a positive HIV diagnosis. The expert clients in the current study had a better understanding of the personal lives of the AGYM they served, which facilitated their appropriate diagnosis of the underlying issues faced by their peers, and their choice of support to provide. Relatedly, expert clients are increasingly being utilized to deliver psychosocial support to newly diagnosed HIV-positive people in several sub-Saharan African countries [31,32]. Lastly, the therapeutic approach (group sessions) used in the present study could have engaged and induced feelings of belonging amongst the CBT beneficiaries, hence enabling free sharing of experiences and support for each other to overcome the fears surrounding a positive diagnosis [33]. On the other hand, literature highlights that expert clients require additional resources, skills, and training to improve their work, especially in the realm of psychosocial support [27].

Furthermore, our findings are consistent with a growing body of evidence on the feasibility and acceptability of lay counselor-delivered counselling interventions in low- and middle-income countries. For example, group-based community health workers delivered counselling for depressed HIV positive patients, which was found to be feasible and acceptable in South Africa [34], and interpersonal therapy (IPT) has also been successfully delivered by CHWs in Pakistan and Uganda [35,36]. Additionally, a systematic review of psychosocial interventions delivered by non-specialists found them to be beneficial to recipients compared to recipients who did not receive any psychosocial treatment at all [16]. Relatedly, psychosocial interventions such as cognitive behavioural therapy (CBT), problem-solving therapy (PST), psycho-education, and IPT have been increasingly found to be feasible and effective within high-income countries and LMICS [37-39]. However, literature emphasizes that lay counselor-delivered therapies should be provided within parameters of adequate training and supervision from a specialist [17,34,40], which leads to capacity building in addition to motivation for continuing work.

Our study contributes to the body of knowledge regarding peer-led interventions for stigma reduction among AGYM living with HIV in resource-limited settings. The tested peer-led group CBT model highlights the critical role that can be played by expert clients in increasing access to psychosocial support for marginalized sub-populations who cannot afford, and have limited access to, psychosocial support by professional psychologists or mental health counsellors. The primary strength of the paper lies in the use of a quasi-experimental pre-and post-test design with a control arm. The use of a control group helped to statistically adjust for confounding variables. Furthermore, the multisite (four health facilities) nature of the study design facilitated stronger external validity. Important to note are the limitations of this study. First, the study relied on self-reported information, and information bias is likely to have occurred due to over- or under-reporting. Second, as a facility-based model, we are unaware of how it would fare in an uncontrolled environment (e.g. a community). Therefore, further studies are required to address the latter. Thirdly, the purposive facility selection might have introduced some bias because factors like differences in health facility capacity, availability of partner organizations, proximity to referral hospitals, and existing psychosocial support services could influence patient experiences and outcomes independently of the CBT intervention. The parallel trends assumption in DiD is theoretical and untestable because of no multiple pre-treatment observations, which is a known limitation of two-period DiD designs. Lastly, although training and supervision were provided to the expert clients, the study did not formally measure intervention fidelity or inter-facilitator variability, both of which are important areas for future research. Despite the limitations, this study presents important insights into the potential of the expert client-delivered CBT in reducing HIV self-stigma, hence informative for HIV-related psychosocial programming.

**Strengths and limitations of this study:** i) the use of a quasi-experimental pre-and post-test design with a control arm. The control group supported the statistical adjustment for confounding variables; ii) the DiD estimated the effect of the intervention (expert client delivered group CBT) by comparing the changes in outcomes over time between the intervention group and the control group; iii) multisite (four health facilities) nature of the study design facilitated stronger external validity; iv) information bias is likely to have occurred in the form of over- or under-reporting of stigma elements.

## Conclusion

In this study, the DiD analysis demonstrated that the expert client delivered group CBT intervention showed a significant effect on the reduction in HIV self-stigma between baseline and endline. The results also showed that the expert client delivered the CBT model, which significantly impacted the reduction of the individual stigma indicators- internalized stigma, *disclosure*, self-image, and public attitudes. These findings provide strong evidence that peer-delivered group CBT is an effective intervention for reducing HIV self-stigma and should be considered for integration and scale-up within routine HIV care programs in resource-limited settings, where trained psychologists and mental health counselors are in short supply, and psychosocial services are expensive.

### What is known about this topic

- Cognitive-behavioural therapy (CBT) can reduce internalized (self-) stigma and improve mental health outcomes, but most evidence comes from specialist-delivered interventions;
- Pregnant adolescent girls and young mothers face compounded vulnerabilities, youth, poverty, and limited youth-friendly services, which heighten self-stigma and hinder engagement across the HIV care continuum.

### What this study adds

- This study offers granular evidence on the practicability of training and supervising lay “expert clients” to facilitate structured group CBT aimed at reducing HIV self-stigma in PMTCT/EID clinics, thus broadening access to psychosocial support in areas with limited mental health specialist services;
- The study demonstrates that incorporating peer-led CBT into standard HIV services for underserved AGYM is feasible and has potential for expansion; it also emphasizes the importance of continuous supervision and quality assurance to maintain the outcomes.
